# Regulation of GluA1 AMPA Receptor Through PKC Phosphorylation Induced by Free Fatty Acid Derivative HUHS2002

**DOI:** 10.1007/s11745-012-3736-4

**Published:** 2012-11-02

**Authors:** Takaaki Nishimoto, Takeshi Kanno, Tadashi Shimizu, Akito Tanaka, Tomoyuki Nishizaki

**Affiliations:** 1 Division of Bioinformation, Department of Physiology, Hyogo College of Medicine, 1-1 Mukogawa-cho, Nishinomiya, 663-8501 Japan; 2 Laboratory of Chemical Biology, Advanced Medicinal Research Center, Hyogo University of Health Sciences, 1-3-6 Minatojima, Chuo-ku, Kobe, 650-8530 Japan

**Keywords:** HUHS2002, GluA1, Modulation, PKC, Phosphorylation

## Abstract

The present study investigated the effect of 4-[4-(*Z*)-hept-1-enyl-phenoxy] butyric acid (HUHS2002), a newly synthesized free fatty acid derivative, on α-amino-3-hydroxy-5-methyl-4-isoxazole propionic acid (AMPA) receptor responses. HUHS2002 potentiated currents through GluA1 AMPA receptors expressed in *Xenopus* oocytes in a bell-shaped concentration (1 nM–1 μM)-dependent manner, the maximum reaching nearly 140 % of original amplitude at 100 nM. The potentiation was significantly inhibited by GF109203X, an inhibitor of protein kinase C (PKC), but not KN-93, an inhibitor of Ca^2+^/calmodulin-dependent protein kinase II (CaMKII). HUHS2002 had no potentiating effect on currents through mutant GluA1 AMPA receptors with replacement of Ser831, a PKC/CaMKII phosphorylation site, by Ala. In the in situ PKC assay using rat PC-12 cells, HUHS2002 significantly enhanced PKC activity, that is suppressed by GF109203X. Overall, the results of the present study show that HUHS2002 potentiates GluA1 AMPA receptor responses by activating PKC and phosphorylating the receptors at Ser831, regardless of CaMKII activation and phosphorylation.

## Introduction

The AMPA receptor plays a pivotal role in excitatory synaptic transmission. So far, four AMPA receptor subunits such as GluA1, GluA2, GluA3, and GluA4 subunits have been cloned, and the receptors are composed of tetramer containing a solitary subunit or various combinations of the subunits. Of AMPA receptors GluA1/GluA2 receptor is preferentially expressed in the brain. The GluA1 subunit contains several phosphorylation sites. CaMKII phosphorylates at Ser831 on the GluA1 subunit, causing an enhancement in GluA1 AMPA receptor responses [1–6]. In addition, evidence has pointed to Ser831 phosphorylation on the subunit by PKC [7]. PKA, alternatively, phosphorylated at Ser845 on the GluA1 subunit, causing an enhancement in the receptor responses [7, 8].

In our earlier study, the *cis*-unsaturated free fatty acid arachidonic acid enhanced currents through Ca^2+^-permeable AMPA receptors via a CaMKII pathway [9]. 8-[2-(2-Pentyl-cyclopropylmethyl)-cyclopropyl]-octanoic acid (DCP-LA), a linoleic acid derivative, potentiated AMPA receptor responses by indirectly activating CaMKII due to protein phosphatase 1 (PP1) inhibition [10]. These findings suggest that *cis*-unsaturated free fatty acids or their derivatives are capable of potentiating AMPA receptor responses in a CaMKII-dependent manner. An established pathway is that *cis*-unsaturated free fatty acids interact with PKC [11]. We have provided direct evidence for DCP-LA-induced selective and direct activation of PKC-ε [12, 13]. Then, we wondered whether other free fatty acid derivatives exert their actions similar to DCP-LA. To address this question, we have synthesized the free fatty acid derivative 4-[4-(*Z*)-hept-1-enyl-phenoxy] butyric acid (HUHS2002). We have obtained the data that HUHS2002 has the potential to inhibit PP1 activity, thereby indirectly activating CaMKII [14]. HUHS2002 potentiated α7 acetylcholine (ACh) receptor responses in a CaMKII-dependent manner, regardless of PKC or PKA [14].

The present study was conducted to see the effect of HUHS2002 on AMPA receptor responses and the underlying mechanism. We show here that HUHS2002 potentiates GluA1 receptor responses by activating PKC and in turn, phosphorylating the receptor at Ser831.

## Materials and Methods

### Animal Care

All procedures have been approved by the Animal Care and Use Committee at Hyogo College of Medicine and were in compliance with the National Institutes of Health Guide for the Care and Use of Laboratory Animals.

### Synthesis of HUHS2002

To a solution of *p*-hydroxybenzaldehyde (2.0 g, 16.4 mmol) and ethyl 4-bromobutyrate (3.6 ml, 19.7 mmol) in DMF (12 ml) was added potassium carbonate (2.7 g, 19.7 mmol) at room temperature. After being stirred for 8 h at 80 °C, the reaction mixture was added to water. The aqueous layer was extracted with ethyl acetate, and the combined organic layers were dried over anhydrous MgSO_4_, and concentrated under reduced pressure. The crude product was purified by silica gel column chromatography (*n*-hexane/ethyl acetate = 4/1) to give ethyl 4-(4-formyl-phenoxy) butyrate (3.1 g, 81 %) as a colorless oil. ^1^H NMR (400 MHz, CDCl_3_) δ 1.26 (t, *J* = 7.1 Hz, 3H), 2.15 (tt, *J* = 7.0 and 7.0 Hz, 2H), 2.53 (t, *J* = 7.0 Hz, 2H), 4.10 (t, *J* = 7.0 Hz, 2H), 4.16 (q, *J* = 7.1 Hz, 2H), 6.99 (t, *J* = 8.7 Hz, 2H), 7.83 (t, *J* = 8.7 Hz, 2H), 9.88 (s, 1H).

To a solution of *n*-hexyltriphenylphosphonium bromide (434 mg, 1.02 mmol) in THF (2.0 ml) was added a 1.0 M THF solution of sodium hexamethyldisilazide (0.914 ml, 0.914 mmol) slowly at −40 °C under the nitrogen atmosphere. The reaction mixture was stirred for 1 h at −40 °C and 4-(4-formyl-phenoxy)butyrate (200 mg, 0.846 mmol) in THF (1 ml) was added at −70 °C. After being stirred for 1 h at −60 °C, the reaction mixture was added with a saturated aqueous solution of ammonium chloride. The aqueous layer was extracted with ethyl acetate, and the combined organic layers were dried over anhydrous MgSO_4_, and concentrated under reduced pressure. The crude product was purified by silica gel column chromatography (*n*-hexane/ethyl acetate = 10/1) to give ethyl 4-[(4-(*Z*)-hept-1-enyl)phenoxy]butyrate (70 mg, 27 %) as a colorless oil. ^1^H-NMR (400 MHz, CDCl_3_) δ 0.87 (t, *J* = 7.1 Hz, 3H), 1.25 (t, *J* = 7.1 Hz, 3H), 1.26–1.36 (m, 4H), 1.39–1.45 (m, 2H), 2.10 (tt, *J* = 7.3 and 7.0 Hz, 2H), 2.29 (ddd, *J* = 7.4, 7.0 and 7.0 Hz, 2H), 2.53 (t, *J* = 7.3 Hz, 2H), 4.00 (t, *J* = 7.0 Hz, 2H), 4.13 (q, *J* = 7.1 Hz, 2H), 5.55 (ddd, *J* = 11.5, 7.4 and 7.4 Hz, 1H), 6.31 (d, *J* = 11.5 Hz, 1H), 6.83 (d, *J* = 6.8 Hz, 2H), 7.19 (d, *J* = 6.8 Hz, 2H).

To a solution of ethyl 4-[(4-(*Z*)-hept-1-enyl)phenoxy]butyrate (66 mg, 0.216 mmol) in dioxane (2 ml) was added a 1.0 M aqueous solution of lithium hydroxide (0.430 ml, 0.430 mmol) under ice-cooling. After being stirred for 4 h at room temperature, the reaction mixture was added to 1 M aqueous solution of HCl. The aqueous layer was extracted with diethylether, and the combined organic layers were dried over anhydrous MgSO_4_, and concentrated under reduced pressure. The crude product was purified by silica gel column chromatography (*n*-hexane/ethyl acetate = 6/1) to give 4-[(4-((Z)-hept-1-enyl)phenoxy)]butyric acid (HUHS2002) (55 mg, 93 %) as a white solid. ^1^H NMR (400 MHz, CDCl_3_) δ 0.89 (t, *J* = 7.1 Hz, 3H), 1.24–1.38 (m, 4H), 1.38–1.51 (m, 2H), 2.10 (tt, *J* = 7.3 and 7.0 Hz, 2H), 2.29 (dddd, *J* = 7.4, 7.0, 7.0 and 1.4 Hz, 2H), 2.59 (t, *J* = 7.3 Hz, 2H), 4.03 (t, *J* = 7.0 Hz, 2H), 5.56 (ddd, *J* = 11.5, 7.4 and 7.4 Hz, 1H), 6.32 (dd, *J* = 11.5 and 1.4 Hz, 1H), 6.85 (d, *J* = 6.8 Hz, 2H), 7.20 (d, *J* = 6.8 Hz, 2H): ESI-HRMS (negative ion, sodium formate) calculated for C_17_H_23_O_3_ ([M-H]-) 275.1653; found 275.1645.

### In-vitro Transcription and Translation

mRNAs coding the GluA1 subunit were synthesized by in-vitro transcription. For the mutant GluA1 subunit, Ser831 on the GluA1 mRNA was replaced by Ala [mGluA1(S831A)]. Mature *Xenopus* oocytes were surgically removed from female frogs under ether anesthesia and manually separated from the ovary. Collagenase (0.5 mg/ml) treatment was carried out to remove the follicular cell layer, and 24 h later oocytes were injected with approximately 50 nl of mRNAs (1 mg/ml) for the GluA1 subunit or the mGluA1(S831A) subunit, and incubated in Barth’s solution [in mM: 88 NaCl, 1 KCl, 2.4 NaHCO_3_, 0.82 MgSO_4_, 0.33 Ca(NO_2_)_2_, 0.41 CaCl_2_, and 7.5 Tris, pH 7.6] at 18 °C.

### Two-Electrode Voltage-Clamp Recording

Oocytes were transferred to a recording chamber 2–3 days after injection of each subunit mRNA and continuously superfused at 22 °C in a standard extracellular solution (in mM: 88 NaCl, 2 KCl, 1.8 CaCl_2_, and 5 HEPES, pH 7.0) or Ca^2+^-free extracellular solution (in mM: 88 NaCl, 2 KCl, 1 EGTA, and 5 HEPES, pH 7.0). Kainate (100 μM) was bath-applied to oocytes for 10 s at 10-min intervals before and after 10 min of treatment with HUHS2002, and kainate-evoked currents were recorded, i.e., the sampling rate was once per 10 min. It has been established that full recovery of desensitization for AMPA receptors examined here is obtained with 10-min washing-out of kainate, based upon previous experiments. In a two-electrode voltage-clamp configuration, whole-cell membrane currents were recorded with a GeneClamp-500 amplifier (Axon Instruments, Inc., Foster city, CA, USA), filtered at 20–50 Hz, and analyzed on a microcomputer using pClamp software (version 6.0.3, Axon Instruments, Inc.). The electrode used, with the resistance of 2–3 MΩ, was filled with 2 M KCl.

### Cell Culture

Rat PC-12 cells, that were obtained from RIKEN Cell Bank (Tsukuba, Japan), were cultured in Dulbecco’s modified Eagle’s medium supplemented with 10 % (v/v) heat-inactivated fetal bovine serum, 10 % (v/v) heat-inactivated horse serum, penicillin (100 U/ml), and streptomycin (0.1 mg/ml) in a humidified atmosphere of 5 % CO_2_ and 95 % air at 37 °C.

### In-situ PKC Assay

PKC activity in PC-12 cells was assayed by the method as previously described [12]. Cells were treated with HUHS2002 in the presence and absence of GF109203X at 37 °C for 10 min in an extracellular solution (in mM: 137 NaCl, 5.4 KCl, 10 MgCl_2_, 5 EGTA, 0.3 Na_2_HPO_4_, 0.4 K_2_HPO_4_, and 20 HEPES, pH 7.2). Then, cells were rinsed with 100 μl of Ca^2+^-free phosphate-buffered saline and incubated at 30 °C for 15 min in 50 μl of the extracellular solution containing 50 μg/ml digitonin, 25 mM glycerol 2-phosphate, 200 μM ATP, and 100 μM synthetic PKC substrate peptide (Pyr-Lys-Arg-Pro-Ser-Gln-Arg-Ser-Lys-Tyr-Leu) (Peptide Institute Inc., Osaka, Japan). The supernatants were collected and boiled at 100 °C for 5 min to terminate the reaction. An aliquot of the solution (20 μl) was loaded onto a reversed phase high performance liquid chromatography (HPLC) (LC-10ATvp, Shimadzu Co., Kyoto, Japan). A substrate peptide peak and a new product peak were detected at an absorbance of 214 nm (SPD-10Avp UV–VIS detector, Shimadzu Co.). It was confirmed that each peak corresponds to non-phosphorylated and phosphorylated substrate peptide in the analysis of matrix-assisted laser desorption ionization time of flight mass spectrometry (Voyager DE-STR, PE Biosystems Inc., Foster city, USA). Areas for non-phosphorylated and phosphorylated substrate peptide were measured (total area corresponds to concentration of substrate peptide used here), and the amount of phosphorylated substrate peptide was calculated. Phosphorylated substrate peptide (pmol/min/cell protein weight) was used as an index of PKC activity.

### Statistical Analysis

Statistical analysis was carried out using Dunnett’s test.

## Results

### HUHS2002 Potentiates Currents Through GluA1 AMPA Receptors

**Fig. 1:**
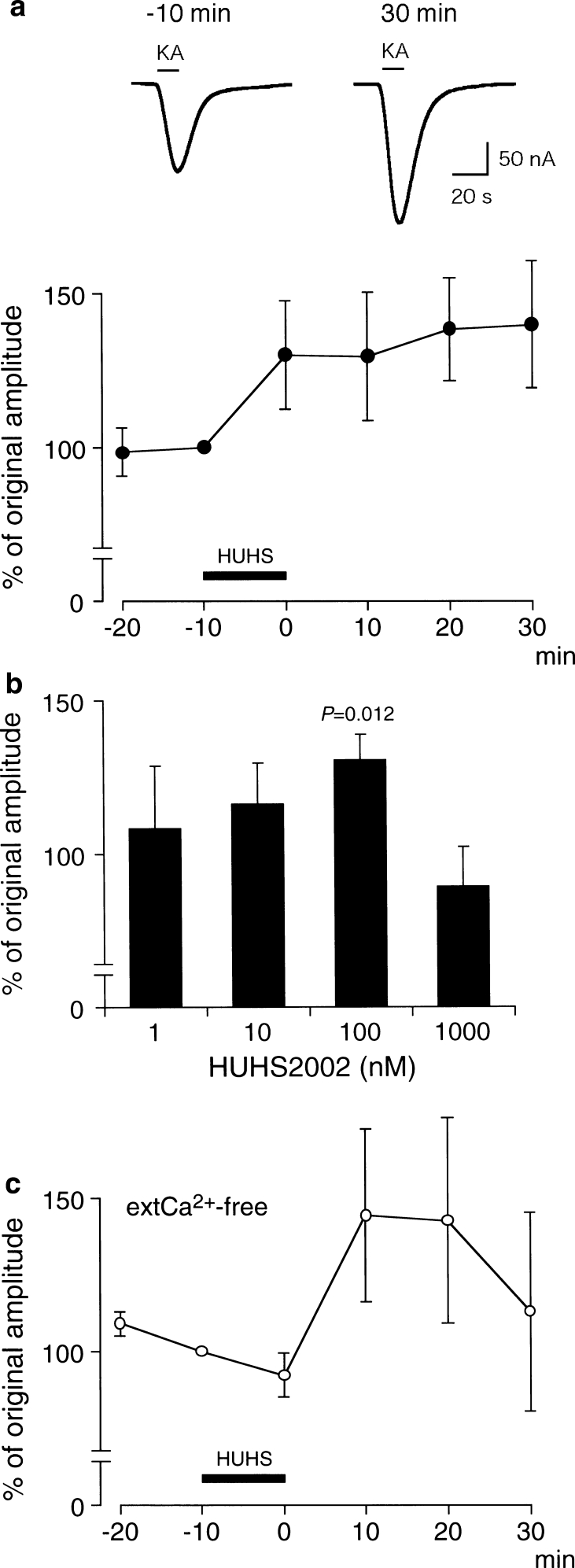
HUHS2002 potentiates GluA1 AMPA receptor currents. **a** GluA1 AMPA receptors were expressed in *Xenopus* oocytes, and kainate (KA) (100 μM) was bath-applied to oocytes for 10 s at a 10-min interval before and after 10-min treatment with HUHS2002 (HUHS) (100 nM) in Ca^2+^-containing extracellular solution. The holding potential was −60 mV. Application with KA is indicated by *bars*. Typical currents recorded 10 min before and 30 min after treatment with HUHS2002 are shown. In the graph, *each point* represents the mean (±SEM) percentage of original amplitudes (−10 min) (*n* = 5 independent experiments). **b** Kainate (100 μM)-evoked currents were recorded 10 min before and 30 min after 10-min treatment with HUHS2002 at concentrations as indicated. In the graph, *each column* represents the mean (±SEM) percentage of original amplitudes (−10 min) (*n* = 5–7 independent experiments). *P* value as compared with current amplitudes before treatment with HUHS2002, Dunnett’s test. **c** Kainate (100 μM)-evoked currents were recorded before and after 10-min treatment with HUHS2002 (100 nM) in Ca^2+^-free extracellular solution. In the graph, *each point* represents the mean (±SEM) percentage of original amplitudes (−10 min) (*n* = 4 independent experiments)

We initially examined the effect of HUHS2002 on responses of AMPA receptors consisting the GluA1 subunit alone, expressed in *Xenopus* oocytes. Kainate (100 μM), an agonist of AMPA receptors, evoked inward whole-cell membrane currents (Fig. 1a). The amplitude of kainate-evoked currents at each period of recording time as indicated in Fig. 1a was not affected by repetitive application with kainate at 10-min intervals in the absence of HUHS2002 (data not shown), indicating full recovery from GluA1 AMPA receptor desensitization. HUHS2002 (100 nM) potentiated kainate-evoked whole-cell membrane currents to nearly 140 % of original amplitude, the effect being evident 30 min after 10-min treatment (Fig. 1a).

The potentiating effect of HUHS2002 was still obtained in Ca^2+^-free extracellular solution (Fig. 1c), indicating that HUHS2002-induced potentiation of GluA1 AMPA receptor currents is due to an enhancement in GluA1 AMPA receptor currents but not in Ca^2+^-sensitive chloride channel currents.

HUHS2002 potentiated GluA1 AMPA receptor currents in a bell-shaped concentration (1 nM–1 μM)-dependent manner, the maximal potentiation being obtained at 100 nM (Fig. 1b).

### HUHS2002 Potentiates GluA1 AMPA Receptor Currents in a PKC-Dependent Manner

**Fig. 2:**
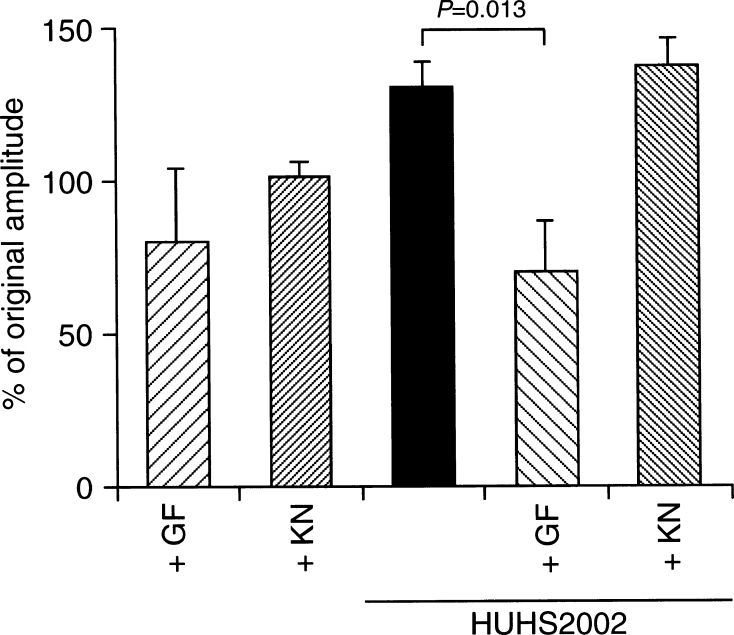
HUHS2002 potentiates GluA1 AMPA receptor currents in a PKC-dependent manner. Kainate (100 μM)-evoked currents were monitored 10 min before and 30 min after 10-min treatment with HUHS2002 (100 nM) in the absence and presence of GF109203X (GF) (100 nM) or KN-93 (KN) (3 μM). In the graph, *each column* represents the mean (±SEM) percentage of original amplitudes (−10 min) (*n* = 4 independent experiments). *P* values, Dunnett’s test

HUHS2002-induced potentiation of GluA1 AMPA receptor currents was significantly inhibited by GF109203X (100 nM), an inhibitor of PKC, to an extent similar to that for the currents elicited from oocytes untreated with HUHS2002 in the presence of GF109203X, but otherwise it was not affected by KN-93 (3 μM), an inhibitor of CaMKII (Fig. 2). This suggests that HUHS2002 potentiates GluA1 AMPA receptor responses in a PKC-dependent manner.

**Fig. 3:**
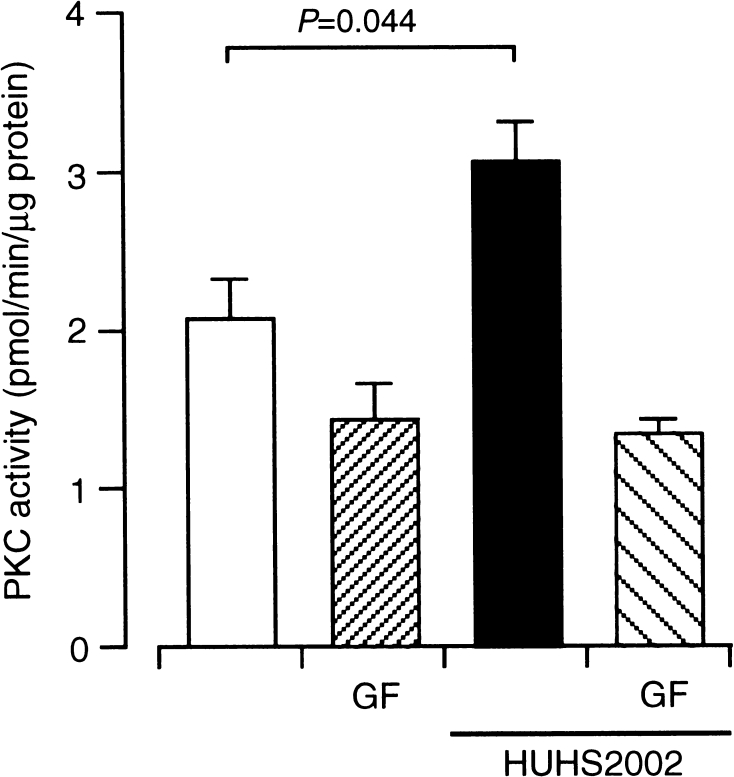
HUHS2002 activates PKC in PC-12 cells. Cells were untreated and treated with HUHS2002 (1 μM) in the presence and absence of GF109203X (GF) (100 nM). Phosphorylated substrate peptide (pmol/min/μg cell protein) was used as an index of PKC activity. In the graph, *each column* represents the mean (±SEM) PKC activity (*n* = 8 independent experiments). *P* value, Dunnett’s test

To obtain evidence for HUHS2002-induced PKC activation, we assayed PKC activity in PC-12 cells. HUHS2002 (1 μM) significantly enhanced PKC activity, and the enhanced PKC activity was reduced by GF109203X (100 nM), to an extent similar to that for cells untreated with HUHS2002 in the presence of GF109203X (Fig. 3). This provides evidence that HUHS2002 is capable of activating PKC.

### HUHS2002 Potentiates GluA1 AMPA Receptor Currents by Phosphorylating the Receptor at Ser831

**Fig. 4:**
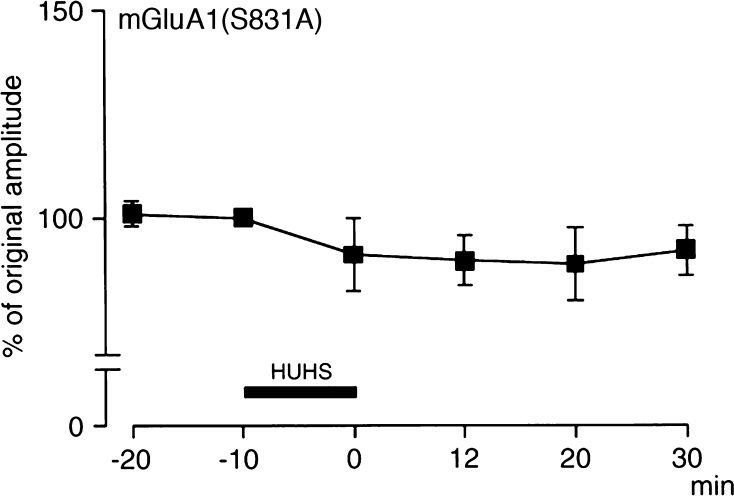
HUHS2002 potentiates GluA1 AMPA receptor currents through PKC phosphorylation of the receptor. mGluA1 (S831A) AMPA receptors were expressed in *Xenopus* oocytes. Kainate (100 μM)-evoked currents for the receptor were monitored before and after 10-min treatment with HUHS2002 (100 nM). In the graph, *each point* represents the mean (±SEM) percentage of original amplitudes (−10 min) (*n* = 4 independent experiments)

It is recognized that Ser831 on the GluA1 subunit is phosphorylated by PKC/CaMKII [1–7]. To see whether HUHS2002-induced potentiation of GluA1 AMPA receptor currents is due to receptor phosphorylation following PKC activation, we constructed mutant GluA1 lacking CaMKII/PKC phosphorylation site [mGluA1(S831A)]. HUHS2002 exhibited no potentiating effect on currents through mGluA1(S831A) receptors (Fig. 4). This indicates that HUHS2002 activates PKC, thereby phosphorylating GluA1 AMPA receptors at Ser831 to potentiate the receptor currents.

## Discussion

The results of the present study clearly demonstrate that the free fatty acid derivative HUHS2002 potentiates GluA1 AMPA receptor responses. In explanation of this, one might point to the implication of CaMKII in the HUHS effect. CaMKII modulates properties of AMPA receptors containing the GluA1 subunit through GluA1 phosphorylation [1, 2] or CaMKII stimulates delivery of AMPA receptors towards the membrane surface, causing an increase in the AMPA receptor conductance [10]. However, this is unlikely here, since HUHS2002-induced potentiation of GluA1 AMPA receptor currents was not affected by KN-93, an inhibitor of CaMKII. We have obtained the data that HUHS2002 indirectly activates CaMKII by inhibiting PP1 [14]. Then, a very complicated question is why HUHS2002, in spite of CaMKII activation, potentiates GluA1 AMPA receptor currents in a CaMKII-independent manner. We have presently no plausible answer and explanation to this.

Our mounting evidence has shown that a variety of *cis*-unsaturated free fatty acids and the linoleic acid derivative DCP-LA activate PKC [12, 13, 15]. In the present study, HUHS2002 enhanced PKC activity in PC-12 cells, that is suppressed by the PKC inhibitor GF109203X, indicating that like other free fatty acids and DCP-LA HUHS2002 could serve as a PKC activator. *cis*-Unsaturated free fatty acids activate novel PKCs including PKC-ε in a Ca^2+^- and diacylglycerol-independent manner or synergistically activate conventional PKCs, possibly by binding the C1 (cysteine-rich) domain [11]. In our earlier study, DCP-LA directly activated PKC-ε under the Ca^2+^-free conditions in the absence of diacylglycerol and phosphatidylserine, and the DCP-LA-induced PKC-ε activation was inhibited by adding phosphatidylserine [12]. This raises the possibility that DCP-LA activates PKC-ε by binding the phosphatidylserine binding site on PKC-ε. HUHS2002, in the light of these facts, might activate PKC by the mechanism sharing with DCP-LA. To address this point, further experiments need to be carried out.

HUHS2002-induced potentiation of GluA1 AMPA receptor currents was inhibited by GF109203X. This, in the light of the fact that the GluA1 subunit contains the CaMKII/PKC phosphorylation site at Ser831 [1–7], implies that HUHS2002 potentiates GluA1 AMPA receptor responses in a PKC-dependent manner. In further support of this note, HUHS2002 had no effect on currents through mGluA1(S831A) AMPA receptors lacking the CaMKII/PKC phosphorylation site.

In conclusion, the results of the present study show that the free fatty acid derivative HUHS2002 potentiates GluA1 AMPA receptor responses by activating PKC and phosphorylating the receptor at Ser831, independently of CaMKII activation and phosphorylation. This may provide further insight into regulation of AMPA receptors by lipids.

## Abbreviations

HUHS2002: 4-[4-(*Z*)-hept-1-enyl-phenoxy] butyric acid
AMPA: α-Amino-3-hydroxy-5-methyl-4-isoxazole propionic acid
PKC: Protein kinase C
CaMKII: Ca^2+^/calmodulin-dependent protein kinase II
DCP-LA: 8-[2-(2-Pentyl-cyclopropylmethyl)-cyclopropyl]-octanoic acid
PP1: Protein phosphatase 1
HPLC: High-performance liquid chromatography
